# Supplementary material for: Modeling glioblastoma heterogeneity as a dynamic network of cell states
Source: Mol Syst Biol. 2021 Sep 16;17(9):e10105. doi: 10.15252/msb.202010105 (PMC8444284; doi:10.15252/msb.202010105)
Supplement: Supplementary file 5 — Source Data for Figure 3 [file MSB-17-e10105-s001.zip › Figure3A_sourcedata/GSEA_3065/nosigdb_state2.GseaPreranked.1622718419215/IVY_CTMVP.html]

Details for gene set IVY\_CTMVP[GSEA]

|  || Dataset | state2 |
| Phenotype | NoPhenotypeAvailable |
| Upregulated in class | na\_neg |
| GeneSet | IVY\_CTMVP |
| Enrichment Score (ES) | -0.7337538 |
| Normalized Enrichment Score (NES) | -2.447635 |
| Nominal p-value | 0.0 |
| FDR q-value | 0.0 |
| FWER p-Value | 0.0 |
Table: GSEA Results Summary

  

Fig 1: Enrichment plot: IVY\_CTMVP      
 Profile of the Running ES Score & Positions of GeneSet Members on the Rank Ordered List

  

| PROBE | GENE SYMBOL | GENE\_TITLE | RANK IN GENE LIST | RANK METRIC SCORE | RUNNING ES | CORE ENRICHMENT || 1 | PDLIM1 |  |  | 452 | 0.190 | -0.0158 | No |
| 2 | GNG11 |  |  | 698 | 0.151 | -0.0163 | No |
| 3 | STEAP1B |  |  | 2362 | 0.052 | -0.1845 | No |
| 4 | ETS1 |  |  | 2998 | 0.034 | -0.2464 | No |
| 5 | RAMP2 |  |  | 3617 | 0.022 | -0.3085 | No |
| 6 | UACA |  |  | 3925 | 0.017 | -0.3383 | No |
| 7 | LAMA4 |  |  | 4068 | 0.014 | -0.3510 | No |
| 8 | PHACTR2 |  |  | 4339 | 0.010 | -0.3781 | No |
| 9 | S1PR3 |  |  | 5237 | -0.004 | -0.4729 | No |
| 10 | NID2 |  |  | 5511 | -0.009 | -0.5005 | No |
| 11 | NID1 |  |  | 5537 | -0.009 | -0.5017 | No |
| 12 | LBH |  |  | 5702 | -0.012 | -0.5172 | No |
| 13 | MCAM |  |  | 5927 | -0.015 | -0.5384 | No |
| 14 | GPX8 |  |  | 6123 | -0.019 | -0.5560 | No |
| 15 | CCDC102B |  |  | 6449 | -0.024 | -0.5865 | No |
| 16 | C1orf54 |  |  | 7100 | -0.039 | -0.6490 | No |
| 17 | KDELR3 |  |  | 7339 | -0.046 | -0.6666 | No |
| 18 | KCNJ2 |  |  | 7552 | -0.052 | -0.6804 | No |
| 19 | ARHGAP18 |  |  | 7679 | -0.056 | -0.6843 | No |
| 20 | A2M |  |  | 7912 | -0.063 | -0.6982 | No |
| 21 | TPM2 |  |  | 8019 | -0.068 | -0.6980 | No |
| 22 | TM4SF1 |  |  | 8150 | -0.074 | -0.6991 | No |
| 23 | ARHGAP29 |  |  | 8476 | -0.094 | -0.7178 | Yes |
| 24 | OLFML2A |  |  | 8569 | -0.101 | -0.7104 | Yes |
| 25 | FILIP1L |  |  | 8586 | -0.102 | -0.6947 | Yes |
| 26 | FRZB |  |  | 8597 | -0.103 | -0.6783 | Yes |
| 27 | TAGLN |  |  | 8662 | -0.108 | -0.6667 | Yes |
| 28 | PCOLCE |  |  | 8685 | -0.110 | -0.6504 | Yes |
| 29 | PRDM1 |  |  | 8693 | -0.110 | -0.6323 | Yes |
| 30 | TGFBI |  |  | 8761 | -0.117 | -0.6195 | Yes |
| 31 | THBS1 |  |  | 8789 | -0.120 | -0.6019 | Yes |
| 32 | COL18A1 |  |  | 8909 | -0.137 | -0.5913 | Yes |
| 33 | TGFBR2 |  |  | 8916 | -0.137 | -0.5686 | Yes |
| 34 | PRSS23 |  |  | 8975 | -0.145 | -0.5501 | Yes |
| 35 | SVIL |  |  | 8981 | -0.146 | -0.5258 | Yes |
| 36 | F2R |  |  | 9026 | -0.153 | -0.5045 | Yes |
| 37 | SERPINH1 |  |  | 9054 | -0.159 | -0.4804 | Yes |
| 38 | COL4A2 |  |  | 9069 | -0.161 | -0.4545 | Yes |
| 39 | FSTL1 |  |  | 9158 | -0.179 | -0.4334 | Yes |
| 40 | COL4A1 |  |  | 9284 | -0.226 | -0.4083 | Yes |
| 41 | LAMC1 |  |  | 9286 | -0.226 | -0.3699 | Yes |
| 42 | IGFBP7 |  |  | 9295 | -0.229 | -0.3318 | Yes |
| 43 | LAMB1 |  |  | 9302 | -0.231 | -0.2932 | Yes |
| 44 | PXDN |  |  | 9312 | -0.240 | -0.2533 | Yes |
| 45 | COL5A2 |  |  | 9316 | -0.244 | -0.2121 | Yes |
| 46 | FN1 |  |  | 9364 | -0.294 | -0.1670 | Yes |
| 47 | STC1 |  |  | 9378 | -0.314 | -0.1151 | Yes |
| 48 | ITGB1 |  |  | 9388 | -0.339 | -0.0584 | Yes |
| 49 | CTGF |  |  | 9400 | -0.375 | 0.0043 | Yes |
Table: GSEA details [plain text format]

  

Fig 2: IVY\_CTMVP: Random ES distribution      
 Gene set null distribution of ES for **IVY\_CTMVP**
